# Optimizing Robotic Mobile Fulfillment Systems for Order Picking Based on Deep Reinforcement Learning

**DOI:** 10.3390/s24144713

**Published:** 2024-07-20

**Authors:** Zhenyi Zhu, Sai Wang, Tuantuan Wang

**Affiliations:** 1 School of Science, Wuhan University of Technology, Wuhan 430070, China; 2 State Key Laboratory of Marine Resource Utilization in South China Sea, Hainan University, Haikou 570228, China; 3 School of Ecology, Hainan University, Haikou 570228, China

**Keywords:** supply chain management, automatic warehousing system, deep reinforcement learning, order allocation and sequencing, robot collaborative scheduling, robotic mobile fulfillment systems, shelf selection

## Abstract

Robotic Mobile Fulfillment Systems (RMFSs) face challenges in handling large-scale orders and navigating complex environments, frequently encountering a series of intricate decision-making problems, such as order allocation, shelf selection, and robot scheduling. To address these challenges, this paper integrates Deep Reinforcement Learning (DRL) technology into an RMFS, to meet the needs of efficient order processing and system stability. This study focuses on three key stages of RMFSs: order allocation and sorting, shelf selection, and coordinated robot scheduling. For each stage, mathematical models are established and the corresponding solutions are proposed. Unlike traditional methods, DRL technology is introduced to solve these problems, utilizing a Genetic Algorithm and Ant Colony Optimization to handle decision making related to large-scale orders. Through simulation experiments, performance indicators—such as shelf access frequency and the total processing time of the RMFS—are evaluated. The experimental results demonstrate that, compared to traditional methods, our algorithms excel in handling large-scale orders, showcasing exceptional superiority, capable of completing approximately 110 tasks within an hour. Future research should focus on integrated decision-making modeling for each stage of RMFSs and designing efficient heuristic algorithms for large-scale problems, to further enhance system performance and efficiency.

## 1. Introduction

With the rapid development of Deep Reinforcement Learning and Machine Learning [1,2], Robotic Mobile Fulfillment Systems (RMFSs) are playing an increasingly important role in the modern logistics field. These systems utilize autonomous mobile robots to perform tasks, such as order picking and goods transportation, aiming to improve logistics efficiency and reduce costs. However, traditional RMFSs often face challenges, such as complex environments, dynamic changes, and task planning limitations, which restrict their performance in practical applications. In this context, the introduction of Deep Reinforcement Learning technology provides new ideas and methods for addressing these challenges.

Deep Reinforcement Learning combines the advantages of deep learning [3,4,5] and reinforcement learning, enabling the learning of complex decision strategies through interaction with the environment and achieving autonomous decision making and intelligent behavior in unknown environments. Therefore, the optimization of RMFSs based on Deep Reinforcement Learning has become a hot topic and focus of current research. Despite the enormous potential of Deep Reinforcement Learning in theory, it still faces a series of challenges in practical applications. Effectively applying Deep Reinforcement Learning technology to RMFSs and solving practical problems is one of the key issues in current research.

In recent years, the swift expansion of the internet has significantly propelled the growth of electronic commerce, consequently elevating consumer expectations for online retailers [6]. The introduction of next-day and even same-day delivery policies has driven related industries to pursue continuous innovation. In the warehousing sector, this necessitates that order processing, picking, and packaging be completed swiftly, to meet the demands of an increasing number of consumers. Traditional warehouses have often struggled to handle the growing volume of orders [7] and have been inefficient in managing small order sizes, including single-line and multi-line orders, as well as a diverse range of products. This has prompted the modernization of warehouses, pushing them towards digitization and automation, to enhance efficiency, accuracy, and operability, thereby making them more adaptable to contemporary business and consumer requirements. Higher efficiency in specific industries translates to greater competitiveness within those sectors [8].

This paper focuses on one such specialized automated system, known as Shelf-Moving Robots, commonly referred to as Robotic Mobile Fulfillment Systems (RMFSs) [9]. RMFSs utilize autonomous robots to perform the picking and sorting tasks of storage shelves (typically referred to as inventory pods or Stock-Keeping Units), thereby eliminating the unnecessary travel and labor associated with human pickers. These robots can autonomously navigate within a warehouse, locate the required shelves, transport the entire shelf to a picking station, and facilitate picking and packaging tasks.

This paper aims to optimize the order-picking process of RMFSs, based on Deep Reinforcement Learning, to improve system efficiency and stability, and to contribute to the development of the modern logistics field.

**Figure 1:**
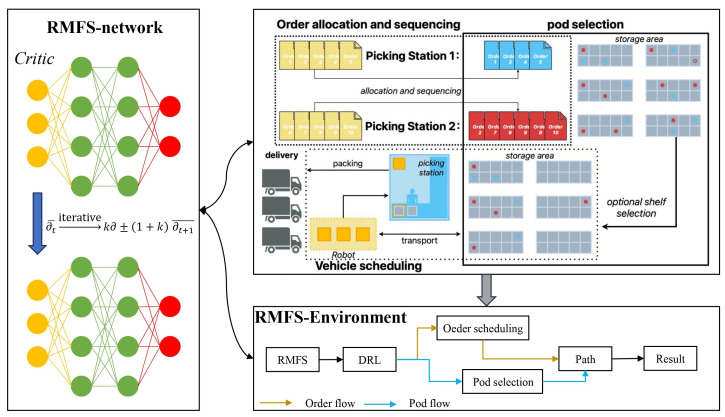
Presentation diagram of optimizing an RMFS for order picking based on DRL.

We investigate the sequential resolution of multiple decision-making challenges within a Robotic Mobile Fulfillment System (RMFS). The process is divided into three phases: order allocation and sequencing, shelf selection, and collaborative robot scheduling. For each phase, we develop distinct mathematical models and employ heuristic algorithms to solve these models in sequence. The diagram illustrating the “Optimization of RMFS for Order Picking Based on Deep Reinforcement Learning (DRL)” is shown below (Figure 1).

The contribution points of this article are as follows:

(1) Intelligent Order Sorting: By integrating the intelligent decision-making capability of Deep Reinforcement Learning into the problem of order allocation and sorting, we have designed an intelligent order-sorting scheme based on Genetic Algorithms. This scheme not only considers factors such as order priority and shelf location but also dynamically adjusts the order allocation sequence based on real-time environmental conditions and the historical experience of the robots, thereby achieving a more intelligent and adaptable order-processing strategy.

(2) Optimization Models for Shelf Transportation: By integrating Deep Reinforcement Learning for complex environments, we have devised 15 optimization models, to minimize shelf transportation distance in diverse warehouse layouts. These models consider warehouse complexities and order intricacies, which are solved using Ant Colony Algorithms. This approach dynamically adjusts, based on real-time data and past experiences, aiding robots engaged in optimal shelf selection and route optimization to tackle varied challenges and find optimal solutions.

(3) Adaptive Robot Scheduling: We have integrated Deep Reinforcement Learning into robot scheduling, proposing an Ant Colony Algorithm for real-time optimization. Robots adaptively learn and refine scheduling strategies, swiftly adjusting to environmental shifts. Our experiments confirm the efficacy of adaptive robot scheduling, which boosts system efficiency and adaptability.

In terms of the structure of the paper, the Section 2 will describe the issues related to the relevant work. In the Section 3, we will introduce our approach, analyzing the key methods, mathematical models, and algorithms. The paper will focus on the decision problem models and solutions in a Robotic Mobile Fulfillment System (RMFS). We formulate mathematical models for the processes of order allocation and sequencing, inventory pod selection, and vehicle scheduling [10,11]. Heuristic algorithms are employed to solve these models separately, addressing these decision problems sequentially.

Regarding order allocation and sequencing, this paper argues that rational allocation and sequencing of orders and order lines can increase the average number of order lines processed per inventory pod, thereby reducing shelf movements and achieving energy and time savings. Sander Teck et al. [12] referred to this optimization as inventory pod consolidation. Additionally, Merschformann et al. [13] noted that another indicator of good performance is a shorter total travel distance. Rational order allocation and sequencing can provide more opportunities for reducing the total travel distance. However, predefined paths suffer from insufficient adaptive self-learning capabilities; so, in this paper a Genetic Algorithm was designed specifically to obtain the order of cargo exit and robot exit. We studied Yanling Zhuang et al. [14], who proposed an efficient mathematical procedure for integrating rack retrieval and relocation in the RMFS environment. Therefore, in this paper an optimization model is established with the objective function of minimizing the travel distance that inventory pods must cover. An Ant Colony Algorithm is utilized to solve this problem, resulting in the allocation and sequencing of inventory pods to various picking stations. Furthermore, due to the interdependencies between order allocation and sequencing with inventory pod selection, this paper proposes that these two decision problems can be further integrated, offering an algorithmic approach to optimizing system performance.

Regarding robot scheduling, since the orders allocated to different picking stations involve different corresponding inventory pods, the travel distance for the robots varies accordingly. Therefore, this paper does not endorse a greedy strategy of immediately transporting the next order to a picking station when it becomes available, as this approach may not result in the shortest overall robot travel time. To address this decision problem [15], this paper develops an Ant Colony Algorithm to determine specific robot scheduling schemes. The experimental results further confirm the effectiveness of this approach, as the algorithm developed in this paper outperforms the greedy algorithm, making robot coordination and delivery more efficient.

This paper sequentially addresses the three decision problems mentioned above. Simulated experiments are conducted, using randomly generated distances between inventory pods and picking stations, distances between inventory pods, and order data. The algorithms developed in this paper exhibit rapid response times for small-scale order data while also being suitable for larger-scale order data.

In the Section 4, we introduce the experimental process, conducting comparative experiments and visualizations, to demonstrate the superiority of our method. The Section 5 discusses our study, exploring the use of Deep Reinforcement Learning (DRL) to optimize the order-picking process in a Robotic Mobile Fulfillment System (RMFS), to improve the efficiency of robotic warehouses. DRL significantly outperforms traditional statistical methods in handling complex, dynamic, and uncertain environments, as it continuously learns to optimize picking strategies. It is particularly suitable for high-dimensional state spaces and warehouse environments requiring complex decisions. We discuss the advantages and disadvantages of the proposed model, highlighting the limitations of our method. These include some constraints in mathematical modeling and algorithm design, such as the lack of solutions for asymmetric problems and limited algorithm adaptability. In the Section 6, we summarize the entire research and provide an outlook for future work.

## 2. Related Work

In a Robotic Mobile Fulfillment System (RMFS), pickers receive order lists at designated picking stations and decide the order sequence at their respective workstations. They select appropriate inventory shelves, collect the items required for the orders, and robots transport these items to the workstations. Each picker processes orders sequentially at their station until all orders are completed. Optimization opportunities in problem analysis primarily lie in order allocation and sequencing, inventory shelf selection, and robot scheduling and coordination [16].

Concerning order allocation and sequencing, research by T. Lamballais et al. suggested that the maximum order throughput is relatively unaffected by the length-to-width ratio of the storage area but is significantly impacted by the placement of the picking stations. Zhan et al. focused on the new trends in omni-channel sales development within the catering industry. They primarily researched how catering businesses can effectively manage their capabilities in key areas, such as order processing, production, and distribution, to meet the needs of customers across different sales channels. Additionally, they applied queuing theory to analyze the characteristics of customer waiting behavior under different delivery modes and the capacity levels of businesses at various stages [17]. Boysen et al. [18] investigated the order-sequencing issue assigned to picking stations, referred to as the Mobile Robot Order Picking Problem (MROP). Their research shows that optimizing the order-picking sequence can halve the number of robots required, thereby enhancing system efficiency. Xie et al. [19] and Valle and Beasley [20] extended Boysen et al.’s model, integrating order allocation and sequencing. However, their mathematical models pay less attention to robot scheduling coordination [21]. The mentioned literature mainly addresses the static aspects of order processing, but order allocation and sequencing can also be performed dynamically, where decision/allocation rules for dynamic sequencing are crucial. To address this, Sander et al. proposed a multi-agent approach for order and vehicle scheduling in RMFSs [22], comparing it with other allocation rules and demonstrating its superiority. Rimélé et al. [23] introduced a mathematical framework that models the operational decisions of RMFSs as a dynamic stochastic program, which assists researchers in devising more advanced and efficient methods [24].

In terms of inventory shelf (rack) selection, Lamballais et al. [25] developed a queuing network model and introduced several key decision variables, such as the number of shelves per SKU (Stock-Keeping Unit) and the replenishment level of each shelf. They concluded that throughput performance can be significantly improved when inventory is distributed across multiple shelves and replenished timely before complete depletion. Yang et al. [26] simultaneously optimized shelf scheduling and picking station order-sequencing problems. They developed a mixed-integer linear programming model and proposed a two-stage heuristic approach to tackling this joint optimization problem. Their experimental results indicated that optimizing both order sequencing and shelf scheduling together can reduce robot tasks by up to 50.8% and 32.0%, respectively, offering valuable managerial insights. Zhuang et al. [27] considered workload balancing among multiple picking stations and shelf-conflict issues, modeling the multi-station sequencing and shelving problems as a mixed-integer programming model. Their approach performed well on both small- and large-scale data, reducing shelf movements by up to 62% compared to current company practices.

Concerning robot scheduling coordination, the work by Roy et al. [28] focused on developing a two-stage stochastic model to analyze different strategies for allocating robots across multiple storage zones. The researchers found that sharing robots across storage zones, rather than dedicating robots to specific zones, could reduce the expected order-picking throughput time by up to two thirds. However, this approach also led to an increase in the expected replenishment time. Chen et al. conducted an in-depth investigation into the optimization of courier dispatch strategies in delivery systems sensitive to customer delays. They performed a detailed analysis from multiple perspectives: comparing dedicated and pooled strategies, introducing a more realistic Poisson demand distribution assumption, exploring the key factors influencing the choice of dispatch strategies, and extending the discussion to more complex scenarios, such as the maximization of social welfare [29]. Yuan and Gong [30] evaluated the performance of an RMFS (Robotic Mobile Fulfillment System) by calculating the throughput time and establishing a queuing network model to determine the optimal number and speed of the robots within the system. Wang et al. [31] employed an open queuing network model to simulate an RMFS (Robotic Mobile Fulfillment System), comparing different layout configurations. Their work provided valuable guidance for determining optimal layout configurations of Robotic Mobile Fulfillment Systems. Sander Teck et al. [32] focused on the integrated-order problem, encompassing both the workstation and robot scheduling. They proposed a two-layer memory algorithm that effectively captured the interdependencies between various aspects of an RMFS (Robotic Mobile Fulfillment System). Additionally, Gharehgozli and Zaerpour [33] emphasized the importance of addressing the scheduling problem in Robotic Mobile order-Fulfillment Systems. They modeled the problem as an asymmetric traveling salesman problem with general precedence constraints, and they developed an adaptive large neighborhood search heuristic to solve large-scale instances [34,35]. Simultaneously, Caiming Xiong, Nishant Shukla, et al. proposed a graph-based framework to represent task-oriented knowledge. They successfully unified the theoretical foundation of pairwise grammar onto an actual robotic platform, achieving robot scheduling [36]. Our work, on the other hand, focuses more on robotic cargo classification and path selection.

Additionally, in literature [37], an in-depth exploration was conducted on how to optimize the use of floor space in industrial warehousing environments, using continuous vertical conveyors. The paper focused on paternoster conveyors, establishing mathematical models and conducting comprehensive analyses to evaluate their performance, in terms of design complexity, throughput, motion resistance, and power consumption. The performance was also compared with other conveyor systems. The researchers provided detailed insights into the three aspects mentioned in this paper. They introduced novel Mixed-Integer Programming (MIP) models for the decision problems at hand. They incorporated access across picking stations, to minimize the total travel distance of the robots. They integrated these decision problems into a single MIP model, optimizing system performance but increasing computational complexity, making computation for large-scale orders challenging. Finally, they highlighted the need for future studies to concentrate on creating effective heuristic approaches tailored to addressing integrated RMFS challenges, particularly when dealing with extensive instances of the problem.

## 3. Methodology

**Figure 2:**
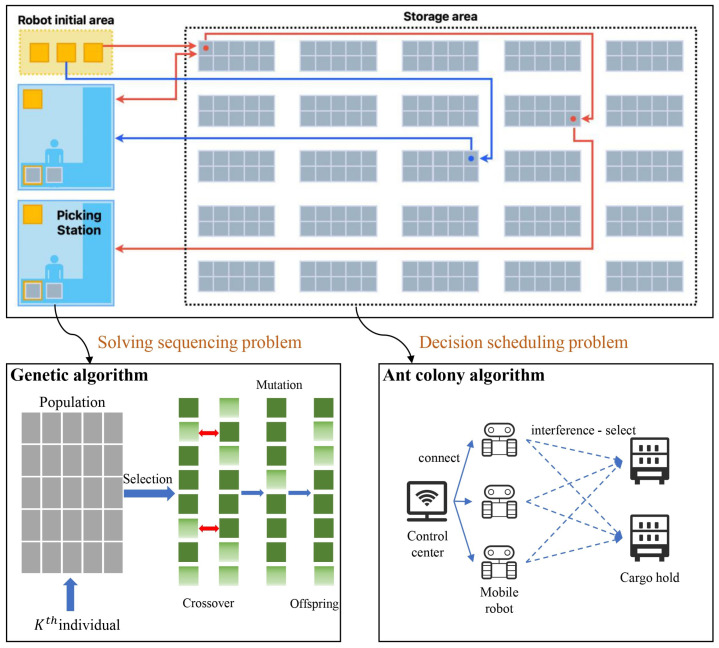
Technical flow of our work with two algorithms.

We focus on sequentially solving multiple decision problems in RMFSs across three stages: order allocation and sequencing, shelf selection, and robot collaborative scheduling. Mathematical models are formulated for each of these three decision problems. Heuristic algorithms are then designed, to solve these models separately in a sequential manner (Technical Flow Figure 2 is shown below).

### 3.1. Key Problem Description

A Robotic Mobile Fulfillment System (RMFS) is a modern warehousing solution designed to enhance the efficiency of warehouse and logistics operations, especially tailored for e-commerce and online retail businesses. In an RMFS, Autonomous Mobile Robots (AMRs) autonomously navigate within the warehouse, locate the required inventory shelves or storage pods, and retrieve the entire shelf, to bring it to a picking station where human pickers carry out the picking and packaging tasks. An RMFS involves a series of interrelated decision problems, including order allocation, order sequencing, inventory pod selection, and robot scheduling [38]. These decision problems are typically interdependent, and optimizing their relationships can significantly improve the performance of the RMFS. The following is a schematic diagram of the three stages involved:

First, regarding order scheduling, we address order allocation and sequencing, using a Genetic Algorithm to obtain the optimal order sequencing. This process includes steps such as population initialization, fitness calculation, selection, crossover, and mutation, to ultimately find the best order allocation and sequence. In an RMFS, optimizing the allocation and sequencing of orders is crucial for enhancing system performance. Imbalanced allocation of order lines can lead to under-utilization of picking stations, resulting in longer picking times. Simultaneously, rational order sequencing can increase the number of order lines processed per inventory pod, thereby improving system efficiency. In this paper, multiple orders are randomly generated and a Genetic Algorithm is employed, to find the optimal order allocation and sequencing. The algorithm design in this paper aims to balance the allocation of orders to picking stations while minimizing the movement of shelves. Due to the interconnections between various optimization problems, optimizing this particular problem ultimately reduces the operational time at picking stations, leading to improved system efficiency.

Second, regarding pod selection, we solve the shelf selection problem, using an Ant Colony Optimization Algorithm, with the objective of minimizing the total travel distance of shelves. Ants select shelves based on pheromone levels, where shorter paths deposit more pheromones, increasing the probability of choosing better shelves. The selection of inventory pods is equally critical for overall optimization. In an RMFS, a mixed-shelve storage strategy is commonly employed, where a specific SKU is distributed across various inventory pods, increasing consolidation opportunities. Typically, picking stations prefer pods that are closer to them, to reduce the total travel distance. However, when a pod can handle multiple orders, choosing that pod may also reduce the total travel distance. Therefore, in some situations, inventory pods located further from the picking station but offering more consolidation opportunities may be more suitable than pods with no consolidation, leading to a decrease in the average distance per picked order line. In summary, optimizing pod selection can also contribute to improving system efficiency.

Third, regarding vehicle scheduling, we solve the robot collaborative scheduling problem, using an Ant Colony Algorithm to determine specific robot scheduling schemes. Idle robots choose the next picking station task based on station completion times and pheromone levels. Better schedules that minimize total processing time have higher pheromone levels in future iterations. In the actual execution of an RMFS, fulfilling picking orders at the picking stations involves a sequence of movements by mobile robots. A robot typically remains at a stationary position until it receives a retrieval task. Subsequently, it moves from its current location to the designated pod location. Upon arrival, the robot can lift the entire inventory pod and transport it to the correct picking station. After the picking process is completed, the robot returns the inventory pod to its storage location and dwells there while waiting for its next task. These movements constitute the operational problem of vehicle scheduling in RMFSs. During multi-robot collaborative scheduling, there may be instances of inefficient waiting at locations like picking stations. Therefore, in some cases, allowing robots to briefly wait then process a pod with a longer processing time may reduce the overall order retrieval throughput time. Proper coordination of robot scheduling can further optimize the system’s efficiency. (The relevant assumptions are shown in Table 1).

### 3.2. Mathematical Models

**Figure 3:**
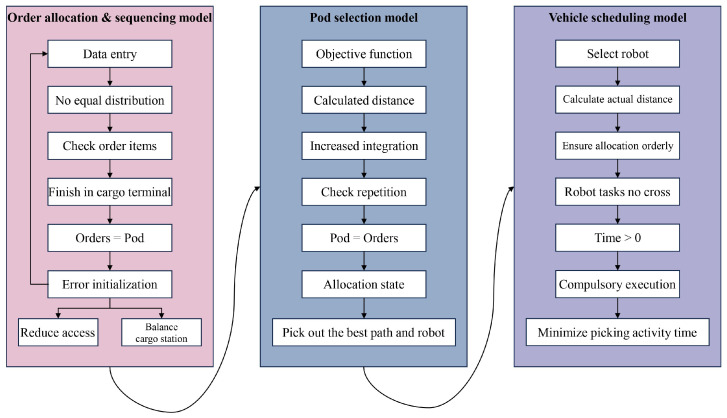
Mathematical model diagram, representing the internal logic of the three mathematical models.

This paper constructs three mathematical models for the sequential optimization of multiple decision problems in Robotic Mobile Fulfillment Systems (RMFSs): the order allocation and sequencing model, the shelf selection model, and the robot collaborative scheduling model. The establishment of these three models was guided by different system efficiency optimization objectives, and heuristic algorithms were designed to solve the corresponding decision problems in a targeted manner. Ultimately, the application of this progressive approach, from mathematical modeling to algorithm design, is key to the gradual performance improvement and optimization of the entire complex collaborative system. The three models each play their part, finally promoting the system forward towards the intended goals of maximized throughput and minimized operating costs (Figure 3).

Before expanding the description of the mathematical model, the meaning of the symbols is shown in the following table (Table 2):

The first mathematical model is the order allocation and sequencing model. In this model, the total distance traveled by robots is closely linked to the total number of robot visits. Simultaneously, achieving a balanced distribution of order lines among picking stations is crucial for minimizing the overall task completion time. Therefore, the objective of this model is to minimize both the number of robot visits and the imbalance of order lines across picking stations. Our mathematical model for the proposed order allocation and sequencing problem is shown below. It is designed to ensure that the allocation of goods is as even as possible, thereby preventing scenarios where a single robot has to travel to multiple distant locations:

(1) $$\min z=\sum_{s\in S}\left(\sum_{i=1}^{n_{s}^{o}}n_{c_{i}^{s}}-B\right)+\sum_{s\in S}n_{s}^{p}$$

(2) $$c_{ij}^{s}\neq c_{ij}^{s},\forall s\in S,\forall i\in \left[1,n_{s}\right],\forall j,j^{\prime }\in \left[1,n_{c_{i}^{s}}\right]$$

(3) $$c_{ij}^{s}\in O_{c_{i}^{s}},\forall s\in S,\forall i\in \left[1,n_{s}\right],\forall j,j\in \left[1,n_{c_{i}^{s}}\right]$$

(4) $$\overset{n_{c_{i}^{s}}}{\underset{j=1}{⋃}}c_{ij}^{s}=O_{c_{i}^{s}},\forall s\in S,\;\forall i\in \left[1,n_{s}\right]$$

(5) $$a_{k}^{s}\in P_{b_{m}^{s}}\forall s\in S,\forall m\in \left[1,n_{s}^{p}\right],\forall k\in \left[n_{m-1}^{s},n_{m}^{s}\right]$$

(6) $$n_{0}^{s}=1,\forall s\in S$$

(7) $$\sum_{s\in S}X_{OS}=1,\forall o\in O$$

(8) $$X_{os}=0\;\mathrm{or}\;1,\forall o\in O,\forall s\in S$$

where $X_{os}$ is whether an order o∈O is allocated to a picking station s∈S; $a_{k}^{s}$ is an item from order line *k* at picking station *s*; $b_{m}^{s}$ is the index of the selected pod *m* at picking station *s*; $c_{i}^{s}$ is order number *i* at picking station *s*; $c_{ij}^{s}$ is the item number of order line *j* from order *i* at picking station *s*; $n_{c_{i}^{s}}$ is the number of order lines in order *i* at picking station *s*; $n_{0}^{s}$ is the number of orders at picking station *s*; $n_{s}^{p}$ is the number of selected pods at picking station *p*; $n_{m}^{s}$ is the sequential order number of the last order line contained in the selected pod *m* at picking station *s*; *B* is the integer variable minimum number of order lines allocated to the picking station; $X_{o}s$ is 1 when order o∈O is allocated to station s∈S or is 0 when otherwise.

The objective function (1) for this **order allocation and sequencing model** aims to minimize the number of visits made by inventory pods, thereby promoting consolidation and ensuring balanced workloads among picking stations.

In this model, both objectives—minimizing visits and balancing picking stations—are equally important, and their relative weights can be adjusted as needed.

- **Constraint (2)**: Ensures identical items from different orders are not assigned to the same picking station.
- **Constraint (3)**: Guarantees that the orders assigned to picking stations match the item requirements of the original orders.
- **Constraint (4)**: Mandates that all orders and order lines are assigned and completed at picking stations.
- **Constraint (5)**: Ensures selected pods align with the order lines.
- **Constraint (6)**: Serves as an initialization condition.
- **Constraint (7)**: Restricts certain decision variables to binary values.

The second mathematical model is the pod selection model. It is worth noting that the model takes as input the order allocation and sorting results computed by the previous mathematical model, thus significantly reducing the search space. However, this ordering approach may lead to sub-optimal solutions compared to integrating these two problems. The mathematical model, which, in practice, ultimately outputs the optimal pod allocation and ordering within each picking station, has the formulae and associated constraints shown below:

(9) $$z=\min\sum_{s\in S}\left[d_{b_{1}^{s}}+2\cdot d_{b_{1}^{s}}^{s}+\sum_{m=2}^{n_{s}^{p}}\left(2\cdot d_{b_{m}^{s}}^{s}+d_{b_{m-1}^{s}b_{m}^{s}}\right)\right]$$

The relevant limitations of the mathematical model algorithm are as follows:

(10) $$c_{ij}^{s}\neq c_{ij}^{s},\forall s\in S,\forall i\in \left[1,n_{s}\right],\forall j,j^{\prime }\in \left[1,n_{c_{i}^{s}}\right]$$

(11) $$c_{ij}^{s}\in O_{c_{i}^{s}},\forall s\in S,\forall i\in \left[1,n_{s}\right],\forall j,j\in \left[1,n_{c_{i}^{s}}\right]$$

(12) $$\overset{n_{c_{i}^{s}}^{s}}{\underset{j=1}{⋃}}c_{ij}^{s}=O_{c_{i}^{s}},\forall s\in S,\;\forall i\in \left[1,n_{s}\right]$$

(13) $$a_{k}^{s}\in P_{b_{m}^{s}},\forall s\in S,\forall m\in \left[1,n_{s}^{p}\right],\forall k\in \left[n_{m-1}^{s},n_{m}^{s}\right]$$

(14) $$n_{0}^{s}=1,\forall s\in S$$

where $a_{k}^{s}$ is the *k*-th order line corresponding to goods at the *s*-th picking station; $b_{m}^{s}$ is the index of the *m*-th selected pod at the *s*-th picking station; $c_{i}^{s}$ is the order number of the *i*-th order at the *s*-th picking station; $c_{ij}^{s}$ is the goods number corresponding to the *j*-th order line in the *i*-th order at the *s*-th picking station; $n_{c_{i}^{s}}$ is the number of order lines in the *i*-th order at the *s*-th picking station; $n_{s}^{o}$ is the number of orders at the *s*-th picking station; $n_{s}^{p}$ is the quantity of pods selected by the *s*-th picking station; $n_{m}^{s}$ is the sequence number of the last included order line in the *m*-th pod selected by the *s*-th picking station.

The objective function (9) of this model aims to minimize the total distance traveled, which includes the distances from the inventory pods to the picking stations and between the pods. This is done to enhance consolidation opportunities. The constraints are similar to the mathematical model above: constraint (10) ensures that there are no duplicate goods assigned to the picking stations; constraint (11) ensures that the orders assigned to the picking stations correspond to the original orders’ goods requirements; constraint (12) requires that all orders and order lines are allocated to the picking stations and completed; constraint (13) ensures that the selected pods correspond to the order lines; constraint (14) serves as an initial condition; finally, constraint (8) ensures that certain decision variables can take binary values.

Lastly, the third mathematical model is the vehicle scheduling model. We present the mathematical modeling for the vehicle scheduling problem. Following the sequential approach, once we have solved the pod assignment problem from the previous section, we will obtain the optimal assignment and ordering of the shelves. These tasks will be used as inputs to the model in this section and assigned to the available mobile robots. In evaluating the accuracy of the model presented in this section, we schedule these tasks to the sorting stations, and these computations will dispatch the robots to load the goods under the fulfilled time conditions with high accuracy and time efficiency. The relevant mathematical formulae and constraints are shown below:

(15) $$\min Z=\max\left(T_{l}^{r}+\frac{D_{l}^{r}}{V_{0}}+2AT\right)$$

The relevant limitations of the mathematical model algorithm are as follows:

(16) $$D_{l}^{r}=d_{e_{(l-1)r}^{p}}e_{lr}^{p}+2\cdot d_{e_{lr}^{p}}^{e_{lr}^{s}},l\neq 0,\;\forall r\in R$$

(17) $$D_{l}^{r}=d_{e_{lr}^{p}}^{p}+2\cdot d_{e_{lr}^{p}}^{e_{lr}^{s}},l\neq 0,\;\forall r\in R$$

(18) $$T_{m}^{s}⩾T_{m-1}^{s}+PT,\forall s\in S$$

(19) $$T_{l}^{r}⩾T_{l-1}^{r}+\frac{D_{l-1}^{r}}{V_{0}}+2AT,\forall r\in R$$

(20) $$T_{0}^{s},T_{0}^{r}=0,\forall s\in S,\forall r\in R$$

(21) $$T_{m}^{s},T_{l}^{r}⩾0,l\in \left[1,N_{r}\right],m\in \left[1,N_{s}\right],\forall s\in S,\forall r\in R$$

where $T_{l}^{r}$ is the start time for the movement of the *r*-th robot, selecting the *l*-th shelf; $T_{m}^{s}$ is the start time for the *s*-th picking station, selecting the *m*-th shelf; $e_{lr}^{p}$ is the shelf number corresponding to the *l*-th task for the *r*-th robot; $e_{lr}^{s}$ is the picking station number corresponding to the *l*-th task for the *r*-th robot; $N_{r}$ is the number of mobile shelves assigned to the *r*-th robot; $N_{s}$ is the number of mobile shelves assigned to the *s*-th picking station.

In the objective function (15), this paper minimizes the completion time of picking station activities. Constraints (16) and (17) are used to calculate the distance traveled by each robot when performing tasks. Constraint (18) ensures that the tasks allocated within a picking station are performed in order, while constraint (19) ensures that the tasks assigned to robots are also performed in order. Constraint (20) is an initial condition. Task start times should be greater than 0, and constraint (21) enforces this property.

### 3.3. Algorithm

**Figure 4:**
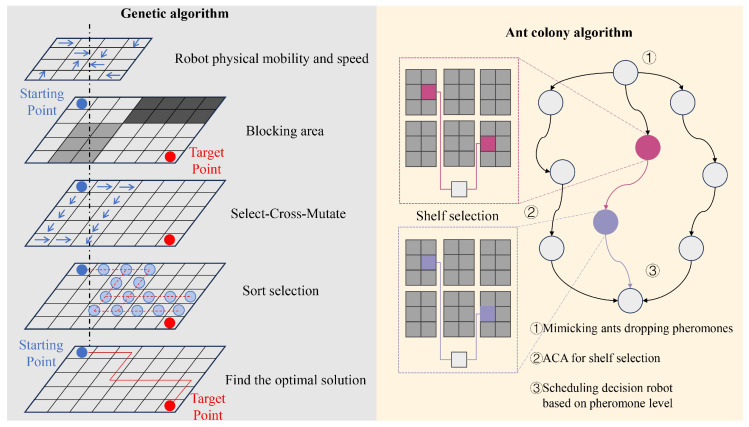
The use of a Genetic Algorithm and an Ant Colony Algorithm in our work.

We use a Genetic Algorithm for solving the order allocation and sequencing problem by searching for the optimal solution through genetic operators, such as selection, crossover, and mutation [39]. Then, an Ant Colony Algorithm is used to solve the shelf selection problem by simulating the behavior of ants dropping pheromones to find the shortest path. Also, the ACO algorithm can be used to solve the collaborative robot scheduling problem, where the ants make scheduling decisions according to pheromone levels. Finally, some basic optimization techniques, such as mathematical modeling, randomization methods, iterative search, etc., are also used. Various heuristic optimization algorithm techniques are used to solve combinatorial optimization problems in the RMFS (Figure 4 shows the details).

The first problem to solve is the order allocation and sequencing problem, the solution specifically referring to the allocation of orders to different picking stations and their respective sequences. In this paper, a Genetic Algorithm is employed to solve the model described above. This includes major steps, such as initializing the population, calculating fitness, selection, crossover, mutation, and obtaining the optimal solution through iterations. In this section, we will focus on explaining how to design algorithms for these key steps.

(1) Population Initialization

In this paper, based on the constraints of the previous model, the pre-generated orders are randomly and non-repetitively assigned to each picking station, while the original order sequence is shuffled, to enhance consolidation opportunities. The pseudocode for generating a feasible solution in Algorithm  1 is as follows:
**Algorithm 1** Generate a Feasible Solution**Input:** latent dimension *K*, knowledge graph *G*, target predicate *p***Output:** $U^{p}$, $V^{p}$, $b^{p}$  1:Construct bipartite subgraph $G_{p}$ from *G* and *p*  2:m← number of subject entities in $G_{p}$  3:n← number of object entities in $G_{p}$  4:Generate training samples $D_{p}$, using uniform sampling  5:Initialize $U^{p}$ (m×K), $V^{p}$ (n×K), $b^{p}$ (n×1) with mean 0 and standard deviation 0.1  6:**for** **all** $\left(s_{p},o_{p}^{+},o_{p}^{-}\right)\in D_{p}$ **do**  7: Update $U_{s}^{p}$, based on Equation (16)  8:**end** **for**  9:**return** $U^{p}$, $V^{p}$, $b^{p}$

Using the above function, multiple initial feasible solutions can be obtained, to form the population needed in this paper.

(2) Calculate Fitness

Regarding fitness calculation, based on the model described earlier, the objective in this problem is to maintain balance among the picking stations and minimize the number of times inventory pods are moved. Calculating the imbalance among picking stations is relatively straightforward. It is important to note that when selecting an inventory pod, it should be capable of handling the current order lines as well as possible subsequent order lines. A smaller value for this objective function is more favorable, resulting in a higher fitness.

(3) Selection

For the selection step, this paper employs a roulette wheel selection method. Individuals with higher fitness values are less likely to be eliminated and have a lower probability of being chosen. Conversely, individuals with lower fitness values are more likely to be eliminated and have a higher probability of being selected. The selection step is primarily implemented in the main function and will not be discussed further here.

(4) Crossover

When an individual is selected, it may undergo a crossover operation. The design of this Algorithm 2 involves crossing the selected individual with the current best individual. Specifically, the order line sequence of a particular order in the selected individual is replaced with the order line sequence of the same order in the best individual. Clearly, this may improve the fitness of the selected individual. Through repeated iterations, we obtain better individuals. The crossover operation function is as follows:
**Algorithm 2** Crossover**Input:** latent dimension *K*, knowledge graph *G*, target predicate *p***Output:** $U^{p}$, $V^{p}$, $b^{p}$  1:Construct bipartite subgraph $G_{p}$ from *G* and *p*  2:m← number of subject entities in $G_{p}$  3:n← number of object entities in $G_{p}$  4:Generate training samples $D_{p}$, using uniform sampling  5:Initialize $U^{p}$ (m×K), $V^{p}$ (n×K), $b^{p}$ (n×1) with mean 0 and standard deviation 0.1  6:**for** **all** $\left(s_{p},o_{p}^{+},o_{p}^{-}\right)\in D_{p}$ **do**  7: Update $U_{s}^{p}$, based on Equation (17)  8:**end** **for**  9:**return** $U^{p}$, $V^{p}$, $b^{p}$

(5) Crossover

When an individual is selected, it may undergo a crossover operation. The design of this Algorithm 3 involves crossing the selected individual with the current best individual. Specifically, the order line sequence of a particular order in the selected individual is replaced with the order line sequence of the same order in the best individual. Clearly, this may improve the fitness of the selected individual. Through repeated iterations, we obtain better individuals. The crossover operation function is as follows:
**Algorithm 3** Mutate**Input:** latent dimension *K*, knowledge graph *G*, target predicate *p***Output:** $U^{p}$, $V^{p}$, $b^{p}$  1:Construct bipartite subgraph $G_{p}$ from *G* and *p*  2:m← number of subject entities in $G_{p}$  3:n← number of object entities in $G_{p}$  4:Generate training samples $D_{p}$, using uniform sampling  5:Initialize $U^{p}$ (m×K), $V^{p}$ (n×K), $b^{p}$ (n×1) with mean 0 and standard deviation 0.1  6:**for** **all** $\left(s_{p},o_{p}^{+},o_{p}^{-}\right)\in D_{p}$ **do**  7: Update $U_{s}^{p}$, based on Equation (12)  8:**end** **for**  9:**return** $U^{p}$, $V^{p}$, $b^{p}$

(6) Overview of Genetic Algorithm

Based on the aforementioned description, the entire Genetic Algorithm 4 iteration can be completed. This includes the main steps of initializing the population, calculating fitness, selection, crossover, and mutation. The result will be the optimal assignment and ordering of orders. The pseudocode for the Genetic Algorithm in this paper is as follows:
**Algorithm 4** Genetic Algorithm**Input:** latent dimension *K*, knowledge graph *G*, target predicate *p***Output:** $U^{p}$, $V^{p}$, $b^{p}$  1:Construct bipartite subgraph $G_{p}$ from *G* using *p*  2:m← number of subject entities in $G_{p}$  3:n← number of object entities in $G_{p}$  4:Generate training samples $D_{p}=\left\{\left(s_{p},o_{p}^{+},o_{p}^{-}\right)\right\}$ via uniform sampling  5:Initialize $U^{p}$ (m×K), $V^{p}$ (n×K), $b^{p}$ (n×1) with mean 0 and standard deviation 0.1  6:**for** **all** $\left(s_{p},o_{p}^{+},o_{p}^{-}\right)\in D_{p}$ **do**  7: Update $U_{s}^{p}$, using Equation (9)  8:**end** **for**  9:**return** $U^{p}$, $V^{p}$, $b^{p}$

The second problem to solve is the pod selection problem. In the solution to the first problem, using the algorithm described above, we obtained the optimal assignment and ordering of orders. In the problem of pod selection, we will use this as input to solve for the optimal pod selection for each picking station. In this problem, for each order line within a picking station, there are three pods to choose from. If the picking station has n order lines, there are $3^{n}$ possible choices. This is clearly an NP-hard problem. Therefore, in this paper, we use an Ant Colony Algorithm to solve this decision problem.

(1) Ant Decision Description

In the Ant Colony Algorithm, a critical aspect is the decision-making process of the ants. In the problem of pod selection, decisions come into play when facing the choice of three pods corresponding to the current order line. Due to the potential for pod consolidation, it is challenging to directly determine which pod selection will have a global optimization effect. Therefore, this paper employs multiple ants that select pods based on the existing pheromone level, leaving behind new pheromone trails. When the total path is shorter, more pheromone is left and, as pheromone accumulates, the probability of choosing that pod also increases. With an increasing number of iterations, the algorithm’s solutions continuously improve, ultimately leading to the optimal solution.

(2) Initialization of Pheromones

To implement this algorithm, the first step is to initialize the pheromones. For each pod, the initialization of pheromones from itself to another pod is set to a constant value. Additionally, when a particular pod is the first one chosen, the pheromone encoding is set from that pod to itself. The pseudocode for initializing pheromones in Algorithm 5 is as follows:
**Algorithm 5** Initialize Pheromone**Input:** blatent dimension *K*, knowledge graph *G*, target predicate *p***Output:** $U^{p}$, $V^{p}$, $b^{p}$  1:Construct bipartite subgraph $G_{p}$ from *G* using *p*  2:m← number of subject entities in $G_{p}$  3:n← number of object entities in $G_{p}$  4:Generate training samples $D_{p}=\left\{\left(s_{p},o_{p}^{+},o_{p}^{-}\right)\right\}$ via uniform sampling  5:Initialize $U^{p}$ (m×K), $V^{p}$ (n×K), $b^{p}$ (n×1) with mean 0 and standard deviation 0.1  6:**for** **all** $\left(s_{p},o_{p}^{+},o_{p}^{-}\right)\in D_{p}$ **do**  7: Update $U_{s}^{p}$, using Equation (12)  8:**end** **for**  9:**return** $U^{p}$, $V^{p}$, $b^{p}$

(3) Updating Pheromones

Updating pheromones is a crucial part of the algorithm’s iterations. In this algorithm, the pheromones are continuously decaying with each iteration. At the same time, new pheromones are added, based on the total distance traveled by each ant. The pseudocode for updating pheromones in Algorithm 6 is as follows:
**Algorithm 6** Update Pheromone**Input:** latent dimension *K*, knowledge graph *G*, target predicate *p***Output:**$U^{p}$, $V^{p}$, $b^{p}$  1:Construct bipartite subgraph $G_{p}$ from *G* using *p*  2:m← number of subject entities in $G_{p}$  3:n← number of object entities in $G_{p}$  4:Generate training samples $D_{p}=\left\{\left(s_{p},o_{p}^{+},o_{p}^{-}\right)\right\}$ via uniform sampling  5:Initialize $U^{p}$ (m×K), $V^{p}$ (n×K), $b^{p}$ (n×1) with mean 0 and standard deviation 0.1  6:**for** **all** $\left(s_{p},o_{p}^{+},o_{p}^{-}\right)\in D_{p}$ **do**  7: Update $U_{s}^{p}$, using Equation (17)  8:**end** **for**  9:**return** $U^{p}$, $V^{p}$, $b^{p}$

(4) Overview of Ant Colony Algorithm

Building upon the functions described above, the design of the entire Ant Colony Algorithm can be completed. In this algorithm, each ant selects a shelf based on the existing pheromone levels, using a roulette wheel selection method to choose the next shelf. Here is the pseudocode for the Ant Colony Algorithm 7 for this problem.

Furthermore, there is a relationship and potential for further integration between the two problems of order allocation and sequencing and of shelf selection. Using the algorithm described above for the order allocation and sequencing problems, it is possible to have multiple solutions with the same objective function value. To further optimize, a straightforward approach is to input these solutions with the same objective values into the algorithm presented in this section. While this is a feasible method, it would increase the algorithm’s time complexity. To integrate both problems effectively, the model can be modified into a multi-objective optimization model, where the solutions would represent order allocation and sequencing, ultimately providing the shelf allocation and sequencing for each picking station.
**Algorithm 7** Ant Colony Algorithm**Input:** latent dimension *K*, knowledge graph *G*, target predicate *p***Output:** $U^{p}$, $V^{p}$, $b^{p}$  1:Construct bipartite subgraph $G_{p}$ from *G* using *p*  2:m← number of subject entities in $G_{p}$  3:n← number of object entities in $G_{p}$  4:Generate training samples $D_{p}=\left\{\left(s_{p},o_{p}^{+},o_{p}^{-}\right)\right\}$ via uniform sampling  5:Initialize $U^{p}$ (m×K), $V^{p}$ (n×K), $b^{p}$ (n×1) with mean 0 and standard deviation 0.1  6:**for** **all** $\left(s_{p},o_{p}^{+},o_{p}^{-}\right)\in D_{p}$ **do**  7: Update $U_{s}^{p}$, using Equation (9)  8:**end** **for**  9:**return** $U^{p}$, $V^{p}$, $b^{p}$

The third problem to solve is the vehicle scheduling problem. In the joint scheduling problem of robots, the algorithm described above uses the shelf allocation and sequencing as its input. There are two fundamental assumptions for this decision problem. First, when scheduling begins, it is evident that available robots should be deployed to fulfill the shelf requirements of picking stations. Second, it is not necessary to schedule robot transportation for the next shelf as soon as there is a vacancy at a picking station. This is because the number of robots is limited, and urgently handling the transportation of shelves for one picking station may lead to excessive waiting times at other picking stations. This paper employs an Ant Colony Algorithm to address this decision problem.

(1) Robot Decision Explanation

When a robot is in an idle state after completing a task, it is assigned to transport the next shelf to a picking station. However, the robot does not directly choose the next available picking station. Instead, it makes its decision based on the time required for each picking station to complete the current picking task and the existing pheromone levels. The probability of a picking station being chosen by a robot is higher when the completion time at that station is shorter and there is more residual pheromone. Additionally, when all picking tasks are completed, new pheromone is deposited, based on the total processing time. Clearly, decisions that lead to shorter total processing times will be favored in the subsequent iterations, resulting in a robot collaborative scheduling plan and minimizing the overall processing time.

(2) Initializing Pheromones

In this problem, the algorithm is designed for two picking stations. Whenever a robot becomes idle, it selects a task from one of the picking stations. When there are more than two picking stations, the initialization of pheromone levels is performed in a similar manner. The pseudocode for initializing pheromones in Algorithm 8 is as follows:
**Algorithm 8** Initialize Pheromone 2**Input:** latent dimension *K*, knowledge graph *G*, target predicate *p***Output:** $U^{p}$, $V^{p}$, $b^{p}$  1:Construct bipartite subgraph $G_{p}$ from *G* using *p*  2:m← number of subject entities in $G_{p}$  3:n← number of object entities in $G_{p}$  4:Generate training samples $D_{p}=\left\{\left(s_{p},o_{p}^{+},o_{p}^{-}\right)\right\}$ via uniform sampling  5:Initialize $U^{p}$ (m×K), $V^{p}$ (n×K), $b^{p}$ (n×1) with mean 0 and standard deviation 0.1  6:**for** **all** $\left(s_{p},o_{p}^{+},o_{p}^{-}\right)\in D_{p}$ **do**  7: Update $U_{s}^{p}$, using Equation (16)  8:**end** **for**  9:**return** $U^{p}$, $V^{p}$, $b^{p}$

(3) Updating Pheromones

Updating pheromones is a crucial part of the algorithm during iterations. Similar to the previous section, in this algorithm, pheromone levels decay with each iteration and new pheromones are added based on the total time taken by each ant. The updating method is similar to the one described earlier, and we will not go into detail here.

(4) Overview of Ant Colony Algorithm

In the algorithm presented in this section, the selection of robot–shelf pairs is also conducted using a roulette wheel method. The pseudocode for this section’s Ant Colony Algorithm 9 is as follows:
**Algorithm 9** Ant Colony Optimization 2**Input:** latent dimension *K*, knowledge graph *G*, target predicate *p***Output:** $U^{p}$, $V^{p}$, $b^{p}$  1:Construct bipartite subgraph $G_{p}$ from *G* using *p*  2:m← number of subject entities in $G_{p}$  3:n← number of object entities in $G_{p}$  4:Generate training samples $D_{p}=\left\{\left(s_{p},o_{p}^{+},o_{p}^{-}\right)\right\}$ via uniform sampling  5:Initialize $U^{p}$ (m×K), $V^{p}$ (n×K), $b^{p}$ (n×1) with mean 0 and standard deviation 0.1  6:**for** **all** $\left(s_{p},o_{p}^{+},o_{p}^{-}\right)\in D_{p}$ **do**  7: Update $U_{s}^{p}$, using Equation (17)  8:**end** **for**  9:**return** $U^{p}$, $V^{p}$, $b^{p}$

**Figure 5:**
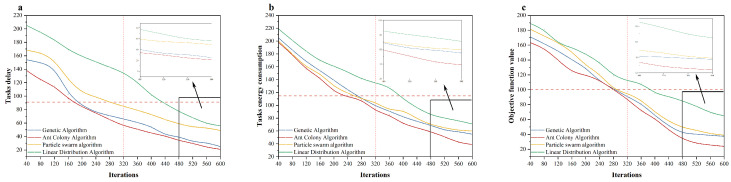
Comparison of four algorithms, in terms of task latency (**a**), task energy consumption (**b**), and function values (**c**).

In order to evaluate the effectiveness of the Genetic Algorithm and the Ant Colony Algorithm, we added a Particle Swarm Algorithm and a Linear Assignment Algorithm to compare with our algorithms. The number of goods was set to 1000 and the number of robots was set to 10. (Figure 5).

## 4. Experiments

### 4.1. Instance Characteristics

**Figure 6:**
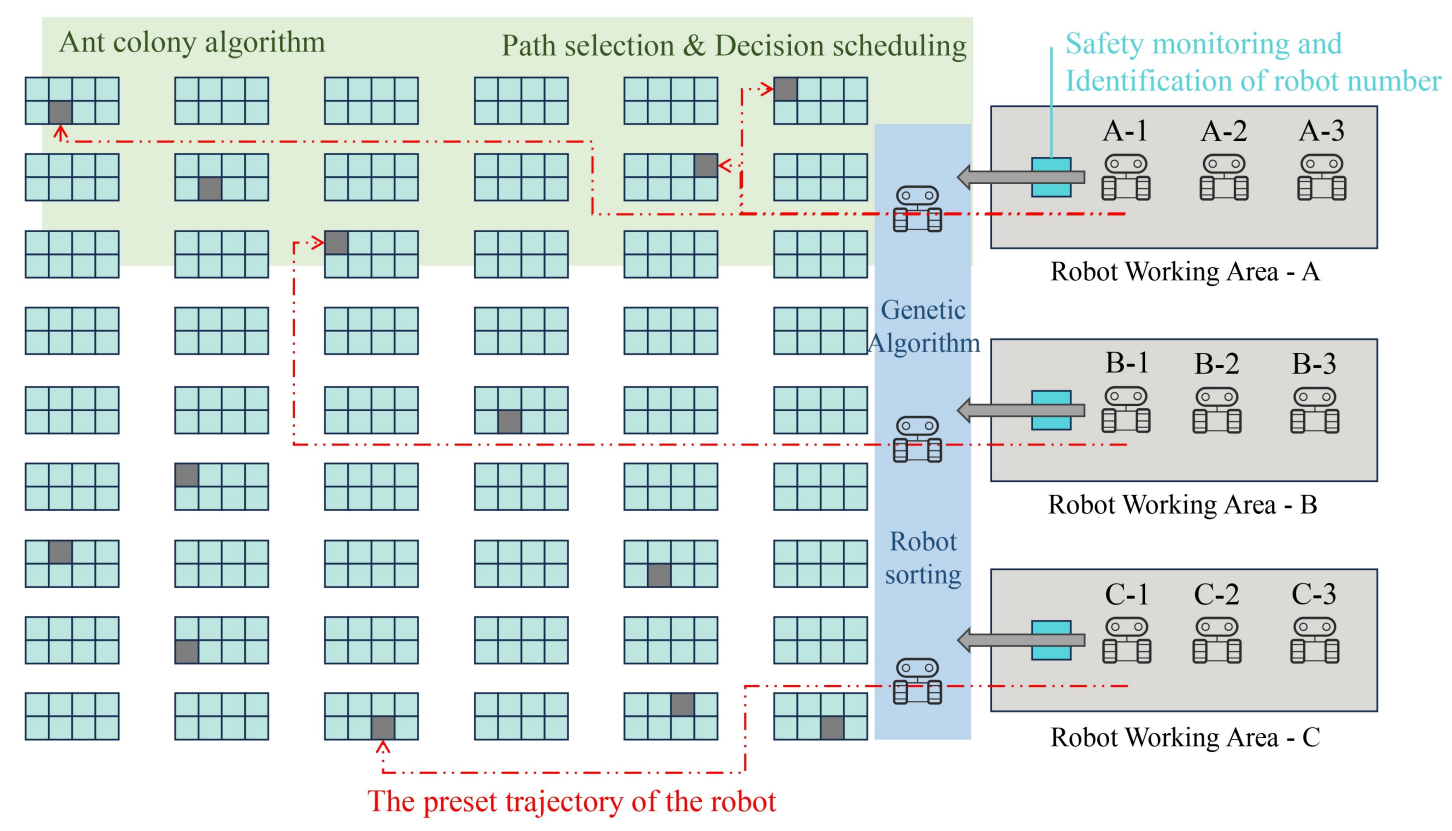
Instance of our robots at work.

First, this paper describes the characteristics of the orders. Larger-scale orders are considered, generating randomly between 100 to 120 orders, with each order containing randomly generated 1 to 3 order lines. In terms of products and shelves, there are 500 types of products, and each type of product can be found on 3 corresponding shelves. There are a total of 300 shelves, and each shelf stores 5 types of SKUs, i.e., products. These data are randomly generated and remain constant in the subsequent problem instances. In terms of distances, the initial positions of the robots are set, and the distance from the initial position to each of the 500 shelves ranges from 1 to 30 m. The distance from each picking station to every shelf is also between 1 and 30 m. The distance between each shelf is 0 to 10 m. These data are also randomly generated and remain constant in the subsequent problem instances. In terms of the performance of robots and picking stations, the robot’s speed is fixed at 1.3 m/s. The time for the robot to lift or lower a shelf is set to 1 s, which has a minor impact on the final results. The picking time for a shelf at a picking station is fixed at 10 s. The following figure shows the order in the cargo hold and the choice of path by multiple robots (Figure 6):

Then, five different layouts of RMFS models are designed, to test the outside approach. Table 3 shows the number of storage stations divided into two groups (large and small): 50 and 300. The size of the model represents the total number of blocks in the model, including tracks, storage stations, and pick station blocks.

### 4.2. Result of a Certain Instance

**Figure 7:**
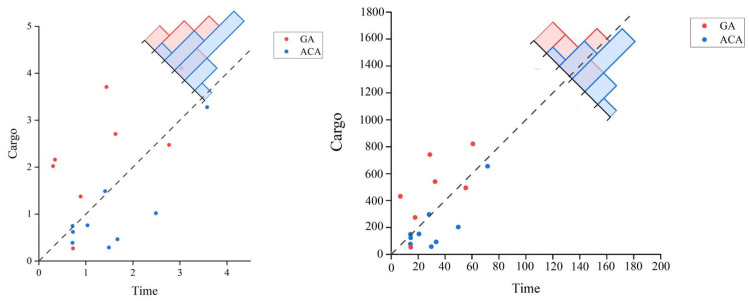
Visual display of time comparison under the execution of the Genetic Algorithm (GA) and the Ant Colony Algorithm (ACA).

The running time of the RMFS algorithm increases linearly with the number of projects. This linearity arises because the algorithm must traverse each item in the list, leading to more iterations as the number of items grows. We conducted experiments with 30 intelligent cargo-moving robots. The results of these experiments, in terms of time and cargo quantity, are depicted in Figure 7.

**Figure 8:**
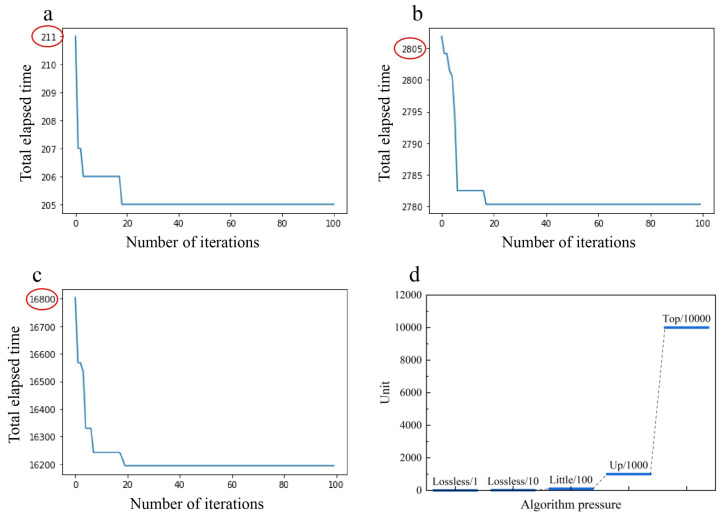
(**a**–**d**) Comparison plot of experimental iterations.

From a test scenario involving 110 orders and a total of 214 order lines, the convergence curves for the objective values during the order allocation and sequencing phases, as well as during the pod selection stages, are shown in Figure 8a,b, respectively. Figure 8c illustrates the variation in total elapsed time relative to the number of iterations during the final stage of collaborative robot scheduling. As observed, the results converge relatively quickly within 100 iterations, underscoring the effectiveness of the proposed algorithm in addressing the problem (Figure 8).

Ultimately, the total time consumed was 2780.3 s, which demonstrates the efficient convergence of the algorithm designed for this stage.

### 4.3. Statistical Data for 30 Instances

Furthermore, the algorithm proposed in this paper processes small-scale orders quite rapidly and remains capable of handling large orders. Our experimental research constraint was 1500 orders with 10 robots. Next, this paper presents statistical data for problem instances with 1000 to 1200 orders (Table 4).

It is important to note that the “Main Distance” here refers to the objective function of the pod selection stage model, specifically the allocation of a robot to transport inventory pods to picking stations. If we wish to obtain the specific distances in the final robot scheduling then they can be calculated by processing the robot scheduling scheme outputted by the algorithm in the robot’s collaborative scheduling stage.

In addition, in the collaborative scheduling stage for robots, the algorithm in this paper, when adjusted to follow a greedy strategy of transporting shelves whenever there is an available picking station, yields inferior results compared to the results obtained by the algorithm proposed in this paper as observed by us. From the above statistics, it can be observed that for around 110 orders, all picking tasks can be completed within one hour. For robot scheduling, we use a dynamic adjustment strategy that allows the algorithm to automatically adapt its decision-making process, based on real-time data. When the order size increases, the algorithm re-evaluates the path planning, to optimize the handling efficiency; when the warehouse layout changes, the algorithm is able to quickly adapt to the new layout, to ensure the continuity of the picking task. To prevent excessive inventory fluctuations leading to robot response errors, we have established a set of exception-handling mechanisms to cope with unforeseen environmental changes. This includes the detection of anomalies, the development of response strategies, and the implementation of recovery plans to ensure that the system can quickly return to normal operation in the face of challenges. This provides reasonable recommendations for the operation of related industries to enhance the efficiency of RMFSs.

## 5. Discussion

Optimizing Robotic Mobile Fulfillment Systems (RMFSs) for order picking using Deep Reinforcement Learning (DRL) focuses on enhancing the efficiency of order picking in robotic warehouses. Analogous to existing robotic warehouses operated by companies like Amazon and Alibaba, mobile robots are employed for goods transportation [40]. However, the proposed approach uniquely emphasizes the application of Deep Reinforcement Learning, to optimize the system’s performance.

Traditional systems typically use collision avoidance algorithms designed based on the kinetic characteristics of mobile robots [41]. However, these algorithms may become less effective over time, due to mechanical aging issues. In contrast, the Cyber–Physical Systems (CPS)-enabled smart robotic warehouse system proposed in this study takes advantage of a digital replica that synchronizes with the real-world environment [42]. This digital counterpart allows for real-time supervision of the physical shop floor and enables predictive analysis of robot motion.

A comparison between DRL and mathematical statistical methods (such as Linear Regression, Cluster Analysis, Decision Trees, and Support Vector Machine) reveals that DRL has distinct advantages in dealing with complex, dynamic, and uncertain environments. This is especially true in scenarios that require robots to learn strategies through trial and error. DRL’s ability to handle high-dimensional state spaces and learn complex decision strategies makes it particularly suitable for environments where loading and unloading goods is complex, or where robots must make decisions in a changing environment. Our actual data set includes not only the 30 instances shown but also scenarios involving up to 15 robots handling 10,000 cargoes, during which DRL demonstrated superior results.

The study does have some limitations concerning mathematical modeling, algorithms, and simulation setup. Compared to Wang et al., who proposed a bottleneck-based model and an open queuing network model, our approach lacks solutions for asymmetric problems [43]. Only a Genetic Algorithm and an Ant Colony Algorithm were considered, leading to limitations in adaptability. Additionally, our constraints are based on laboratory conditions; more rules should be incorporated in future work.

By leveraging Deep Reinforcement Learning, the proposed system optimizes the order-picking process by reducing computation time and alleviating computational burdens [44]. This enables smooth transmission of control commands to mechanical assets, ensuring the effective operation of the system. Unlike reactive approaches that rely on stopping robots to resolve conflicts, the proactive approach employed by the proposed algorithm focuses on finding alternative routes, to avoid collisions [45]. Utilizing computer simulations, the system can effectively supervise the physical environment, predict robot motion, and optimize order-picking operations, ultimately leading to improved efficiency and reliability in RMFSs. Moreover, using Genetic and Ant Colony Algorithms helps in effective path planning and collision prevention, due to concurrent robot path crossings.

Robustness and realism were prioritized in our simulation. First, a simulation environment with multiple warehouse layouts and goods distributions was constructed, to reflect real-world diversity. Key parameters reflecting realistic robot performance were selected and carefully tuned. Additionally, to thoroughly test algorithm effectiveness, a series of scenarios, including peak-hour order processing and emergency order responses, were designed. The DRL algorithm demonstrated excellent adaptability and flexibility, continually optimizing its strategies through learning, to cope with environmental changes. The effectiveness of the method was demonstrated through practical application examples. In large e-commerce warehouses, such as Amazon, the intelligent order-sorting scheme dynamically adjusts order processing sequences based on real-time data, improving picking efficiency. In JD’s unmanned warehouses, the shelf selection optimization model helps robots dynamically adjust shelf selection strategies, reducing transport time and path conflicts. In Tesla’s automated manufacturing plants, the scheduling scheme based on the Ant Colony Algorithm allows robots to optimize scheduling strategies in real time, quickly responding to production line failures and peak demands, and significantly improving system efficiency and stability.

## 6. Conclusions

This paper investigated the sequential resolution of multiple optimization problems in an RMFS. Mathematical models were formulated for three stages: order allocation and sequencing, shelf selection, and robot collaborative scheduling. Heuristic algorithms were designed, to solve each decision problem separately. The algorithm designed in this paper has been applied in practical engineering fields. In large e-commerce warehouses, order allocation and sorting are key to improving picking efficiency. For instance, Amazon’s warehouses process millions of orders daily. By adopting the intelligent order-sorting scheme proposed in this paper, the order processing sequence can be dynamically adjusted based on real-time order data and warehouse layout. For example, high-priority orders and orders located on nearby shelves can be processed first, thereby reducing picking time and energy consumption and improving overall efficiency. In modern logistics centers, such as JD’s unmanned warehouses, robots need to efficiently select and transport shelves in complex warehouse environments. The method presented in this paper can assist these robots in dynamically adjusting shelf selection strategies based on real-time environmental data and historical experience. For example, when a certain area of the warehouse is busy, robots can choose shelves that are closer and less busy, thereby reducing transport time and path conflicts, and enhancing picking efficiency. In automated manufacturing plants, such as Tesla’s production lines, multiple robots need to work collaboratively to complete complex manufacturing tasks. The method proposed in this paper can be used to optimize the scheduling of these robots. For instance, when a workstation on the production line malfunctions, robots can quickly adjust their scheduling strategies, based on real-time data, to avoid production line stoppages and restore normal operations in the shortest time possible. Moreover, during peak periods, robots can dynamically adjust task allocations, to prevent resource waste and workload accumulation.

Due to the interdependencies among different decision problems in an RMFS, integrating the three stages may offer more optimization opportunities compared to the sequential resolution method. In the study on algorithm efficiency, researchers explored an integrated exact algorithm for the three stages and demonstrated that integration significantly improves overall system efficiency. However, they also noted that integration introduces additional computational workload, making their algorithm practical for small-scale orders but impractical for large-scale orders. Therefore, in future research, we can extend the intelligent order-sorting scheme based on genetic algorithms to explore multi-objective optimization problems, such as simultaneously optimizing picking time, energy consumption, and order accuracy. By introducing multi-objective optimization algorithms, we can achieve a balance among various performance metrics. Future studies should also enhance the system’s real-time decision-making capabilities in highly dynamic and uncertain environments by combining Deep Reinforcement Learning with online learning techniques, thereby improving the system’s robustness and responsiveness. In large-scale multi-robot systems, coordination and conflict avoidance between robots are crucial research directions. Future research could explore collaborative scheduling problems in distributed multi-agent systems. Integrating warehouse layout design with path planning for joint optimization will enhance the overall efficiency and flexibility of the system. Additionally, incorporating advanced sensing technologies, such as LiDAR, cameras, and RFID, will improve the robots’ ability to perceive complex environments. Through long-term, large-scale practical system deployment and evaluation, we can continuously optimize system performance, ensuring the method’s long-term effectiveness and sustainability in real-world environments. 

## Figures and Tables

**Table 1:** Simplified assumptions for mathematical modeling, heuristic algorithm development, and result analysis.

Number	Assumption
1	Shelves are initially filled and always have sufficient units to satisfy order picks.
2	Order data are deterministically generated prior to optimization.
3	Each SKU is distributed across various inventory pods, following a mixed-shelve storage policy.
4	Shelves have fixed positions within the storage area.
5	Rare events like robot breakdowns and battery issues are not considered.
6	Robots travel at a constant speed.
7	Robot congestion or collisions are not considered, as lanes are unidirectional and unloaded. Robots can travel beneath the pods, avoiding shared lanes with loaded robots.
8	Robots remain at their service completion location and are pooled rather than dedicated to specific picking stations.
9	Picking times and robot lifting times are fixed.

**Table 2:** Mathematical model symbol meaning.

Mathematical Models	Mathematical Symbols	Explanation of Meaning
Order allocation and sequencing model and Pod selection model	*O*	Set of orders
	$O_{i}$	Set of items belonging to order *i*
	*P*	Set of inventory pods
	$P_{i}$	Set of items belonging to inventory pod *i*
	*S*	Set of picking stations
	$d_{p}$	The initial distance from the starting point to the *p*-th pod
	$d_{pp^{\prime }}$	The distance from the *p*-th pod to the $p^{\prime }$-th pod
	$d_{p}^{s}$	The distance from the *s*-th picking station to the *p*-th pod
Vehicle scheduling model	*R*	Set of picking robots
	PT	Picking time
	AT	Activity time (including lifting and dropping, etc.)
	$V_{0}$	Constant robot velocity

**Table 3:** Comparison with the optimal solution in large size and small size.

	Storage Stations (Large Size, 300)					Optimal Time	Average Time	Percentage Difference
Layout	A	B	C	D	E	67.5 s	75.6 s	89.2%
Cargo of stations	30	35	40	45	50			
Size of model	13 × 13	13 × 13	13 × 13	13 × 13	13 × 13			
Layout type	Rectangular	Rectangular	Fishbone	Fishbone	Fishbone			
Exploring rate	0.8	0.7	0.7	0.8	0.8			
	Storage stations (small size, 50)							
Layout	a	b	c	d	e	2335.2 s	2798.6 s	83.4%
Cargo of stations	30	35	40	45	50			
Size of model	13 × 13	13 × 13	13 × 13	13 × 13	13 × 13			
Layout type	Rectangular	Rectangular	Fishbone	Fishbone	Fishbone			
Exploring rate	0.8	0.7	0.7	0.8	0.8			

**Table 4:** Summarizes the main contents of 30 data points.

Serial Number	Number of Orders	Number of Order Lines	Number of Moving Shelves	Main Distance	Total Time	Unit Time
1	1006	204	201	15,168.2	2583.7	2.6
2	1014	239	233	18,692.8	3162.07	3.1
3	1018	241	239	18,889.8	3203.7	3.1
4	1009	218	211	15,908.1	2767.5	2.7
5	1003	207	204	15,801.3	2679.9	2.7
6	1000	187	182	14,299.1	2444.2	2.4
7	1015	241	234	18,756.8	3130.2	3.0
8	1000	187	186	14,965.6	2465.0	2.4
9	1018	219	216	16,693.4	2858.3	2.8
10	1017	234	229	18,084.3	3062.2	3.0
11	1006	206	203	15,455.1	2677.2	2.6
12	1007	204	195	16,353.0	2749.2	2.7
13	1019	226	219	17,755.7	3028.7	2.9
14	1015	233	225	17,901.4	3023.5	2.9
15	1014	213	209	16,492.1	2800.2	2.7
16	1009	217	211	16,788.9	2841.2	2.8
17	1019	236	231	19,059.4	3192.1	3.1
18	1005	201	197	15,822.1	2667.2	2.6
19	1010	224	220	17,571.0	2938.2	2.9
20	1011	216	209	16,108.0	2729.1	2.7
21	1011	215	208	16,010.1	2731.3	2.7
22	1008	216	213	16,922.3	2867.0	2.8
23	1009	213	206	16,550.9	2811.8	2.7
24	1019	231	224	18,135.1	3009.2	2.9
25	1019	239	233	18,710.0	3131.1	3.0
26	1002	195	191	14,816.8	2519.0	2.5
27	1017	251	244	19,497.2	3319.0	3.2
28	1017	231	225	17,673.2	2980.8	2.9
29	1013	214	209	16,529.1	2802.4	2.7
30	1010	214	208	16,193.5	2780.3	2.7

## Data Availability

Data are contained within the article.
